# An analog of psychedelics restores functional neural circuits disrupted by unpredictable stress

**DOI:** 10.1038/s41380-021-01159-1

**Published:** 2021-05-25

**Authors:** Ju Lu, Michelle Tjia, Brian Mullen, Bing Cao, Kacper Lukasiewicz, Sajita Shah-Morales, Sydney Weiser, Lindsay P. Cameron, David E. Olson, Lu Chen, Yi Zuo

**Affiliations:** 1 Department of Molecular, Cell and Developmental Biology, University of California Santa Cruz, Santa Cruz, CA USA; 2 Departments of Neurosurgery, Neuropsychiatry and Behavioral Sciences, Stanford Neuroscience Institute, Stanford University School of Medicine, Stanford, CA USA; 3 Neuroscience Graduate Program, University of California, Davis, Davis, CA USA; 4 Department of Chemistry, University of California, Davis, One Shields Avenue, Davis, CA USA; 5 Department of Biochemistry & Molecular Medicine, School of Medicine, University of California, Davis, Sacramento, CA USA; 6 Center for Neuroscience, University of California, Davis, Davis, CA USA

**Keywords:** Neuroscience, Psychiatric disorders

## Abstract

Psychological stress affects a wide spectrum of brain functions and poses risks for many mental disorders. However, effective therapeutics to alleviate or revert its deleterious effects are lacking. A recently synthesized psychedelic analog tabernanthalog (TBG) has demonstrated anti-addictive and antidepressant potential. Whether TBG can rescue stress-induced affective, sensory, and cognitive deficits, and how it may achieve such effects by modulating neural circuits, remain unknown. Here we show that in mice exposed to unpredictable mild stress (UMS), administration of a single dose of TBG decreases their anxiety level and rescues deficits in sensory processing as well as in cognitive flexibility. Post-stress TBG treatment promotes the regrowth of excitatory neuron dendritic spines lost during UMS, decreases the baseline neuronal activity, and enhances whisking-modulation of neuronal activity in the somatosensory cortex. Moreover, calcium imaging in head-fixed mice performing a whisker-dependent texture discrimination task shows that novel textures elicit responses from a greater proportion of neurons in the somatosensory cortex than do familiar textures. Such differential response is diminished by UMS and is restored by TBG. Together, our study reveals the effects of UMS on cortical neuronal circuit activity patterns and demonstrate that TBG combats the detrimental effects of stress by modulating basal and stimulus-dependent neural activity in cortical networks.

## Introduction

Stress, often caused by unpredictable and unpleasant events and circumstances, pervades modern life. Acutely, stress elicits adaptive physiological and behavioral responses through which the organism maintains physiological stability, a process termed “allostasis” [1]. However, prolonged stress may overwhelm the capacity of the adaptive mechanisms, causing allostatic overload and predisposing the individual to diseases, particularly mental illnesses [2–5]. Previous rodent studies have revealed the deleterious effects of stress at synaptic, circuit, and behavioral levels. Stress leads to profound changes in dendritic morphology and dendritic spines in a brain region-specific manner [6–8]. For example, chronic stress causes dendritic atrophy and spine loss of pyramidal neurons (PyrNs) in hippocampal CA1 and CA3 areas [9–11], the medial prefrontal cortex [7, 12–14], and the somatosensory cortex [15, 16]. In contrast, it increases spine density on pyramidal and stellate neurons in the basolateral amygdala [17, 18], as well as dendritic branching of PyrNs in the orbitofrontal cortex [14]. At the circuit level, stress disrupts the excitation-inhibition balance and hence affects neural circuit function [15, 19–22]. As a consequence of such synaptic and circuit defects, behavioral abnormalities arise. The literature abounds with reports that chronic stress elevates anxiety and aggression [17, 23], impairs sensory processing [15, 16], and deteriorates decision-making and cognitive flexibility [14, 24].

The pleiotropic effects of stress pose a significant challenge to finding effective therapies. One class of candidate drugs are psychedelics, which have potent and fascinating effects on the human mind by inducing states of altered perception, mood, and thought [25]. Since early attempts to model mental illnesses with mescaline, psychedelics such as (5*R*,8*R*)-(+)-lysergic acid-*N*,*N*-diethylamide (LSD), psilocybin, and *N,N*-dimethyltryptamine (DMT) have attracted much research interest as potential treatments for mental illnesses [26]. Such interest revived after a three-decade hiatus due to restrictive regulations, leading to a plethora of clinical studies testing psychedelics’ therapeutic values in diseases including obsessive compulsive disorder [27], anxiety [28, 29], depression [30–32], and substance abuse [33, 34], with encouraging efficacy outcomes.

Although classical psychedelics appear efficacious in treating stress-induced psychiatric disorders, their hallucinogenic potential remains a significant drawback [35]. Recently, tabernanthalog (TBG), an analog of the psychedelic 5-methoxy-*N,N*-dimethyltryptamine (5-MeO-DMT), was synthesized and found not to induce the head-twitch response in mice [36], a rodent behavioral proxy for hallucinations [37]. TBG exhibits encouraging anti-addictive and antidepressant potential, but its effects on the stressed brain and the underlying neural mechanisms are unknown. In this study, we show that a single dose of TBG given after the stress period rescues various stress-induced behavioral deficits, including anxiety, defective sensory processing, and reduced cognitive flexibility. TBG promotes regrowth of excitatory neuron dendritic spines lost during stress, enhances whisking-modulation of neuronal activity in the somatosensory cortex, and rescues the texture-specific neuronal responses in a texture discrimination task.

## Materials and methods

### Experimental animals

*Thy1*-GFP-M (JAX #007788) and C57BL/6J (JAX #000664) mice were purchased from the Jackson Laboratory. Mice were group-housed with littermates and maintained on a 12 h light/dark cycle. Both sexes of mice aged 1–2 months were used. Unless otherwise noted, all experiments were carried out on C57BL/6J mice. Mice were randomly assigned to experimental groups. All animal experiments were carried out in accordance with protocols approved by the IACUC of University of California Santa Cruz or by Stanford University Administrative Panel on Laboratory Animal Care.

### Unpredictable mild stress

We subjected 2-month old mice to 7-day UMS as previously described [38]. Briefly, the mice were exposed to mild stressors as listed in Supplementary Table 1.

### Elevated plus maze (EPM)

EPM test was performed according to established protocols [39] with slight modifications. We used a custom-made plexiglass EPM. The four arms were 30 cm × 5 cm (L × H); the two closed arms were enclosed by walls 15 cm in height. The apparatus was elevated 50 cm from the ground by sturdy metal posts. Each mouse was allowed to explore the EPM freely for 5 min. Mouse behavior was monitored with a video-tracking system controlled by Bonsai [40]. We used DeepLabCut [41] to track multiple points on the mouse (nose, head, neck, body, and base of the tail) through all video frames, and used custom-written Python 3.6 and Matlab R2019a (MathWorks, Natick, MA) programs to quantify the total distance traveled and the time spent in open vs. closed arms, based on the location of the body point.

### Four-choice odor discrimination and reversal

We followed the protocol described previously [42] with slight modifications. The four-chamber arena is a 12” × 12” × 9” (L × W × H) box constructed of 0.25” white acrylic, with 4 quadrants partially divided by 3”-wide internal walls made of transparent acrylic. White ceramic ramekins (diameter = 2.88”, depth = 1.75”) were used to present odor stimuli and food reward. The odor stimuli were essential oils (rosemary, clove, thyme, nutmeg, or cinnamon; LorAnn Oils, Lansing, MI). Food rewards were small pieces (~10 mg each) of Honey Nut Cheerio (General Mills, Minneapolis, MN). Digging media were made of pine shavings (Grreat Choice®, www.petsmart.com). Between trials, the mouse was confined by a removable transparent acrylic cylinder (diameter = 6”) in the center of the arena. The arena was wiped with 70% ethanol between animals.

The mouse was food restricted starting ~5 days before the testing day so that its body weight was reduced to 80–85% of the baseline. Meanwhile it was also handled 10 min daily for 3 days, followed by a two-day pre-training procedure before the testing day. On pre-training day 1 (acclimation), the mouse was habituated to the arena and the ramekins (one in each quadrant) for 1 h. Food rewards were placed in all ramekins without digging medium coverage. On pre-training day 2 (shaping), the mouse learned to dig in the media to find buried food reward. Only one ramekin was used in this phase, and the quadrant containing it was rotated between trials (SE to NW to SW to NE), with all quadrants rewarded equally. In the first 4 trials, the cereal piece was not covered with wood shaving. Over the next trials the amount of wood shavings gradually increased, from a dusting of shavings (4 trials) to quarter full (4 trials), half full (4 trials), and finally to full coverage of the cereal piece (12 trials). Trials were untimed, and most mice completed shaping within 1 h.

On the testing day, the mouse was subjected to a four-choice discrimination session followed by a reversal session. Each quadrant of the arena contained one ramekin filled with wood shavings and scented by applying a drop of essential oil onto a small piece of filter paper affixed to the ramekin wall. During the initial discrimination phase, the mouse discriminated among the four odors (rosemary, clove, thyme, and nutmeg) and learned which one was associated with the buried cereal reward. The placement of ramekins was pseudo-randomized such that the same odor was not presented in the same quadrant over two consecutive trials. In each trial, the mouse could freely explore the arena until it started digging in a ramekin (rather than merely sniffing or chewing the shavings). If the mouse made a correct choice, the trial was terminated after it finished eating the food reward; if it made an incorrect choice the trial was terminated after it finished digging. If the mouse did not make any choice within 3 min, the trial was terminated and recorded as an “omission”. After each trial, the mouse was returned to the arena center with cylinder confinement, and ramekins were rearranged and re-baited if necessary. The session criterion was met if the mouse correctly completed 8 out of 10 consecutive trials.

The mouse moved on to the reversal session immediately after passing the discrimination session. All shavings were replaced with fresh shavings, and thyme was swapped out for a novel odor, cinnamon. The session criterion was the same as above.

### Whisker-dependent texture discrimination (WTD)

The WTD test on free-moving mice was performed as previously described [15, 43]. Prior to testing, the subject mouse was habituated to the testing chamber (L × W × H = 38 cm × 28 cm × 23 cm) for 10 min per day for 2 days. On the testing day, the mouse went through habituation (3 min), encoding (5 min), resting (5 min), and testing (3 min). During encoding, the mouse was presented with two columns (3 cm × 3 cm × 12 cm) coated with the same texture (e.g., 220 grit sandpaper). During testing the columns were replaced with a new pair, one with the same texture as before (familiar) and the other with a novel texture (e.g., 60 grit sandpaper), for the mouse to explore. We excluded mice showing insufficient interest in the columns (i.e., <12 total approaches) or biased towards one column (i.e., >60% approaching time spent on one column) during encoding from further testing.

The WTD test on head-fixed mice was performed using the Neurotar mobile home cage (MHC; Neurotar Oy Ltd, Helsinki, Finland). The mouse was handled 5–10 min per day for 3 days to habituate it to the experimenter. Then the mouse was habituated to head-fixation on the empty MHC (1 h session × 2 per day for at least 6 days). On the testing day the mouse went through 4 phases: free exploration (5 min), encoding (15 min), resting (10 min), and testing (15 min). During encoding, the mouse was presented with two identical textures (e.g., 5 cm × 5 cm patches of 220 grit sandpaper attached to the MHC wall, separated by 90 degrees) to explore. During resting, the textures were removed and the mouse was allowed to rest or explore the empty MHC at will. During testing, two fresh textures were attached to the MHC wall, one identical to the texture previously presented, the other novel (e.g., 60 grit sandpaper). Mouse behavior was recorded with an infrared camera (Cameleon 3 monochrome CM3-U3-13Y3M-CS, FLIR Systems, Inc., Richmond, BC, Canada) and analyzed offline using the Boris program [44].

We quantified the number of approaches and the amount of time spent actively investigating the columns, and computed the discrimination index (DI) as follows:

$${\it{DI}} = \frac{{{\mathrm{approaches}}\,{\mathrm{to}}\,{\mathrm{novel}}\,{\mathrm{texture}} - {\mathrm{approaches}}\,{\mathrm{to}}\,{\mathrm{familiar}}\,{\mathrm{texture}}}}{{{\mathrm{approaches}}\,{\mathrm{to}}\,{\mathrm{both}}\,{\mathrm{textures}}}}$$

### Drug preparation and administration

TBG was synthesized in the lab of David E. Olson as described previously [36]. TBG or fluoxetine hydrochloride (Cat #0927-10, Tocris Bioscience, Bristol, U.K.) was administered to the mouse intraperitoneally (i.p.) at a dosage of 10 mg/kg of bodyweight. USP-grade saline (0.9%) was used as vehicle.

### Cranial window implantation and virus injection

We performed cranial window implantation and virus injection on mice around postnatal day (P)30 according to established protocols [45] with slight modifications. In brief, the mouse was anesthetized with isoflurane (4% for induction, 1.5% for maintenance). Ophthalmic ointment was applied to prevent eye desiccation and irritation; dexamethasone (2 μg/g bodyweight) was injected into the quadriceps, and carprofen (5 μg/g bodyweight) was injected intraperitoneally. A circular piece of the skull was removed with a trephine (diameter = 2.3 mm, Fine Science Tools, Foster City, CA) driven by a high-speed micro-drill (Foredom K1070, Blackstone Industries, LLC, Bethel, CT). The window centered at AP = −1.5 mm, ML = 3.5 mm for barrel cortex (S1BF), or AP = +1.7 mm, ML = 1.0 mm for frontal cortex. For dendritic spine imaging, we used the *thy1*-GFP-M line mice, which express cytoplasmic GFP in a sparse subset of cortical neurons [46]. For Ca imaging of cortical L2/3 neurons, we used C57BL/6J mice and injected AAV2/1-Syn-GCaMP6f-WPRE-SV40 (Addgene 100837-AAV1) at two sites (~150 nl per site) near the center of the cranial window using a custom-built injection system based on a single-axis oil hydraulic micromanipulator (Narishige, Tokyo, Japan), targeting 150–200 μm below pial surface. The cranial window was sealed with an imaging port made of a round glass coverslip (#1, diameter = 2.3 mm) glued to an overlaying annular glass “doughnut” (#1, inner diameter = 2 mm, outer diameter = 3 mm, Potomac Photonics, Inc., Baltimore, MD). Dental cement (Jet Denture Repair, Lang Dental, Wheeling, IL) was applied over the exposed skull to secure a custom-made stainless-steel head plate onto the skull. The mouse received the antibiotic enrofloxacin (5 μg/g bodyweight) and the analgesic buprenorphine (0.1 μg/g bodyweight) preemptively and then daily for 2 more days.

### In vivo spine imaging and image analysis

In vivo two-photon (2P) imaging of dendritic spines was performed on a 2P microscope (Ultima Investigator, Bruker Co., Middleton, WI) equipped with a 16× NA = 0.8 water immersion objective (CFI75 LWD 16X W, Nikon Instruments, Inc., Melville, NY) and an ultrafast 2P laser (Mai Tai, Spectra-Physics, Santa Clara, CA) operating at 940 nm. The mouse was anaesthetized with an intraperitoneal injection of a mixture of 17 mg/ml ketamine and 1.7 mg/ml xylazine in 0.9% saline (5.0 ml/kg bodyweight), and mounted on a custom-made stage for imaging. Stacks of images were acquired with a Z-step size of 1 µm at 12× zoom. Relocation of the same dendrites in subsequent imaging sessions was achieved by reference to blood vessels and the dendritic branching pattern. Data analysis was performed in ImageJ as described previously [47, 48]. Typically, 150–200 spines were analyzed per animal per session. The percentage of spines formed/eliminated was calculated as the number of spines formed/eliminated divided by the total number of spines counted from the previous imaging session. Morphological categorization of spines was performed according to criteria described previously [49]. Filopodia were identified as described previously [50]. The percentage of filopodia formed/eliminated was calculated as the number of filopodia formed/eliminated divided by the total number of protrusions (spines plus filopodia) counted from the previous imaging session.

### In vivo wide-field Ca imaging and image analysis

The awake mouse was head-fixed over a custom-made flat rotating disk on which it may run or rest at will. Wide-field Ca imaging through the cranial window was performed on a custom-built mesoscope adapted from a previously published design [51] (see Fig. 3a for a schematic drawing). Briefly, images of the brain surface were taken through a pair of photographic lenses in tandem (focal length 50 mm, F = 1.2 and 5.6, respectively) coupled to a scientific cMOS camera (PCO Edge 5.5, ~6.5 μm pixel resolution; PCO AG, Kelheim, Germany). A blue light-emitting diode (470 nm, max power 1000 mW; Thorlabs #M470L3) provides the excitation light, which passes through a 480/30 nm bandpass filter (Chroma Technology AT480/30x) and is deflected by a dichroic mirror (Chroma Technology T4951pxr). Emitted fluorescence passes through a 520/36 nm bandpass filter (Edmund Optics 67-044) and is detected by the camera. In each imaging session, 16-bit images (400 × 400 pixels) were collected at 10 frames per second (fps) for 15 min. A profile view of the mouse, focusing on the whisker pad contralateral to the cranial window, was collected concurrently with an infrared camera (Raspberry Pi NoIR V2) at 30 fps triggered by the onset of wide-field imaging.

Wide-field Ca imaging data were first processed with pySEAS, an independent component analysis (ICA) filtering method to remove components corresponding to hemodynamic changes; other components were recombined for subsequent data analysis [52]. A mask for the regions with strong virus expression was generating by thresholding the autocorrelation of dF/F_0_ of each pixel (threshold = 0.95). Only pixels in the masked regions were used for subsequent analysis.

To define whisking episodes, we first manually selected a region of interest (ROI) around the whisker pad from the behavioral video in OpenCV. We then used a grid-based optic flow algorithm to calculate the motion magnitude across grid points. We used the average motion magnitude of all grid points to represent the magnitude of whisking.

The whole-field Ca activity Ca_WF_ was defined as the average dF/F_0_ over all pixels in the masked region. Its cross-correlation with whisking magnitude was defined with the Pearson correlation coefficient calculated in Python 3.6 using the *NumPy* function *numpy_correlate*. For trial-by-trial analysis, Ca_WF_ around the onset of each whisking episode (−2 s to +2 s) were extracted and aligned. The whisking-modulation of Ca_WF_ was calculated as follows: first subtract the average pre-onset Ca_WF_ over (−1 s to 0 s) from the average post-onset Ca_WF_ over (0.3 s to 1.3 s) for each episode, then average the results over all whisking episodes for each animal. The response window was chosen based on a previous work [53]. The response delay was calculated as follows: first find the maximum value of post-onset Ca_WF_ over (0.3 s to 1.3 s) for each episode, then average the results over all whisking episodes for each animal. The cross-correlation between whisking magnitude and individual pixel’s dF/F_0_ was computed using the *NumPy* library as above.

### In vivo 2P Ca imaging and image analysis

Imaging was performed with a 2P microscope (Ultima Investigator, Bruker Co., Middleton, WI) equipped with a 16× NA = 0.8 water immersion objective (CFI75 LWD 16X W, Nikon Instruments, Inc., Melville, NY), a resonant scanner, and an ultrafast 2P laser (Mai Tai, Spectra-Physics, Santa Clara, CA) operating at 940 nm. Ca images (512 × 512 pixels) were taken at 150–200 μm beneath the pial surface at 30 fps. Image series were motion-corrected with the “moco” plug-in of ImageJ [54] and then down-sampled to 10 fps by average every three consecutive images. ROIs corresponding to individual neurons were manually delineated from the standard deviation projection image (along the time axis of the series) using ImageJ, and the mean pixel value F for each ROI was extracted. The extracted neuronal Ca traces were analyzed using a custom-written program in Matlab. According to the cell morphology and the appearance of Ca traces, the majority of neurons labeled by AAV2/1-Syn-GCaMP6f-WPRE-SV40 were excitatory, consistent with previously published observations [55]. Putative excitatory neurons with noisy signals and no apparent Ca transient were excluded from further analysis.

To compute dF/F_0_, F_0_ was estimated as the 50th percentile value of F within a 300 s sliding window. Ca transients and their peaks were detected using the Matlab function *peakfinder*, with minimal peak height = 4 × standard deviation of baseline dF/F_0_, minimal inter-peak interval = 0.5 s, and minimal transient width = 0.3 s. The denoised dF/F_0_ was obtained by setting to zeros the values of the trace below 2 × standard deviation of baseline.

Synchronous Ca events was detected and analyzed based on the previously published method [56] with slight modifications. Briefly, we first binarized each neuron’s denoised dF/F_0_ by setting non-zero values to 1 and then summed the binarized Ca traces of all neurons to yield the population Ca trace. We then constructed surrogate population Ca traces (1000 trials) by circularly shuffling each neuron’s binarized Ca trace independently and summing the shuffled traces. The random circular shuffling maintains the average activity level of each neuron. We then found the 95th percentile value of all surrogate population Ca traces across the entire time course as the synchrony threshold. The time point at which the population Ca trace exceeded this threshold was set as the start of a synchronous Ca event, and the time point at which it fell below this threshold was set as the end of the event. A neuron was considered to participate in a synchronous event if it was active at the peak of the synchrony.

To determine the pairwise correlation between neuronal Ca activities, we computed the Pearson correlation coefficient ρ between the dF/F_0_ of each pair of neurons. In order to cluster neurons based on their pairwise correlation, we first transformed ρ into a distance metric $d = \sqrt {(1 - \rho )}$, and then constructed a hierarchical cluster tree based on this distance metric using the Matlab function *linkage* (with ‘complete’ method). We used the clustering results to order the neurons in the correlation matrix plot.

We identified a “touch” event as the period of time lasting more than 1 s, during which whiskers contralateral to the imaged S1BF were in contact with the texture. We excluded shorter periods to ensure that the mouse was not merely passing by the texture unintentionally. The average touch response of a neuron to each texture was calculated as follows: first subtract the average baseline dF/F_0_ (−1 s to 0 s before contact) from the average touch dF/F_0_ (0.3 s to 1.3 s after interaction onset) for each interaction, then average the results over all interactions with the texture. We used receiver operating characteristic (ROC) analysis [57] to identify neurons responding to either novel or familiar texture, or both. We calculated the detection probability (DP), which is the probability with which an ideal observer could predict whether the Ca signal corresponds to a contact or baseline immediately before contact. To do so we split each episode of interaction into the pre-contact period (−1 s to 0 s) and the contact period (0.3 s to 1.3 s after contact onset), and assign the decision variable (DV) on the basis of the neuronal Ca activity (average dF/F_0_ over the period). We then calculated DP as the area under the ROC curve for discrimination on the basis of DV. In order to assess the significance level, we performed a random permutation test, in which Ca activities during pre-contact and contact periods were randomly reshuffled (1000 times), and DP was calculated for each shuffling. We consider a neuron as responsive if *p* < 0.05. If a neuron responds exclusively to the novel or the familiar texture, it is classified as novel texture-selective (NTS) or familiar texture-selective (FTS), respectively. If a neuron responds to both textures, it is classified as non-selective.

### In vitro electrophysiology

To prepare acute brain slices for patch-clamp recording [58], mice (aged 6–8 weeks) were anesthetized with isoflurane and decapitated. The brains were quickly removed and transferred into ice-cold cutting solution containing the following (in mM): 70 NaCl, 2.5 KCl, 1.25 NaH_2_PO_4_, 26 NaHCO_3_, 25 glucose, 75 sucrose, 4 MgCl_2_, and 0.5 CaCl_2_. 300 µm thick coronal slices were made with a vibratome (VT1200, Leica Microsystems, Wetzlar, Germany) in the cutting solution. After cutting, slices were immediately transferred to 32–34 °C artificial cerebrospinal fluid (ACSF) containing the following (in mM): 120 NaCl, 26 NaHCO_3_, 2.5 KCl, 11 glucose, 2 CaCl_2_, 2 MgSO_4_, and 1 NaH_2_PO_4_, (pH 7.3, ~300 mOsm). The ACSF and the cutting solution were balanced with 5% CO_2_ / 95% O_2_. Slices recovered at 32–34 °C for 30 min before incubation in ACSF at room temperature.

Patch-clamp recordings in the whole-cell configuration were performed at room temperature on PV+ INs in S1BF L2/3 (visualized with an Olympus BX51WI microscope). Recording pipettes (3–4 MΩ) were filled with the internal solution (in mM: 130 K-gluconate, 10 KCl, 10 HEPES, 5 MgATP, 0.3 Na_3_GTP, 0.2 EGTA, and 0.2% biocytin; pH 7.3, ~300 mOsm). For membrane property measurements, we broke-in under the voltage-clamp mode, held the cell at −70 mV, and immediately measured membrane resistance and capacitance. Once the cell was stabilized, we gradually reduced the holding current to 0, and then switched to current-clamp mode to measure resting membrane potential and other active membrane properties. The resting membrane potentials were recorded ~15 s after the switch to current-clamp mode. Input resistances were measured by holding the membrane potential at −60 mV. Action potential (AP) discharges and cell excitability were assessed by injections of a series of DC current steps (−50 to +330 pA in 20 pA increment for 800 ms, with 8 s inter-trial-interval) in the absence of any neurotransmitter receptor antagonist. The number of APs elicited by the injected currents was quantified. The rheobases were measured by injecting a series of current at 2 pA increment. Data were acquired using a Multiclamp 700B amplifier, Digidata 1440A, and pClamp10 software (Molecular Devices, San Jose, CA). Sampling rate was 20 kHz. Neurons with >10% changes in *R*_m_, *R*_a_, or *C*_m_ were excluded from further analysis.

### Quantifications and statistical analyses

Choice of sample size was based on studies published previously using similar animal models and experimental paradigms. All behavioral and imaging data were analyzed with the analyst blinded to the experimental conditions. All statistical analyses were performed with GraphPad Prism 8.4 (GraphPad Software, San Diego, CA). We performed the Shapiro–Wilk test for sample normality and examined the homogeneity of variance. Unless otherwise stated, if the sample met the assumptions for parametric tests, we used two-sided unpaired *t*-test for two-sample comparison, and one-way Analysis of Variance (ANOVA) followed by *post hoc* Tukey’s multiple comparisons for multi-sample comparison. If the sample failed to meet the assumptions for parametric tests, we used the Wilcoxon signed rank test for two-sample comparison, and the Kruskal–Wallis test followed by Dunn’s multiple comparisons for multi-sample comparison. We reported the sample sizes in the figures and the statistical tests used in the figure legends. We reported the *p* values of main effects in ANOVA or Kruskal–Wallis test in figure legends, and marked the *p* values of *post hoc* multiple comparisons in the figures with asterisks if they reached statistical significance (*p < 0.05, **p < 0.01, ***p < 0.001, ****p < 1 × 10^–4^); comparisons that did not reach statistical significance were not marked. Data are presented as mean ± s.e.m. unless otherwise stated.

## Results

### Post-stress treatment with a single dose of TBG rescues stress-induced behavioral deficits

**Fig. 1:**
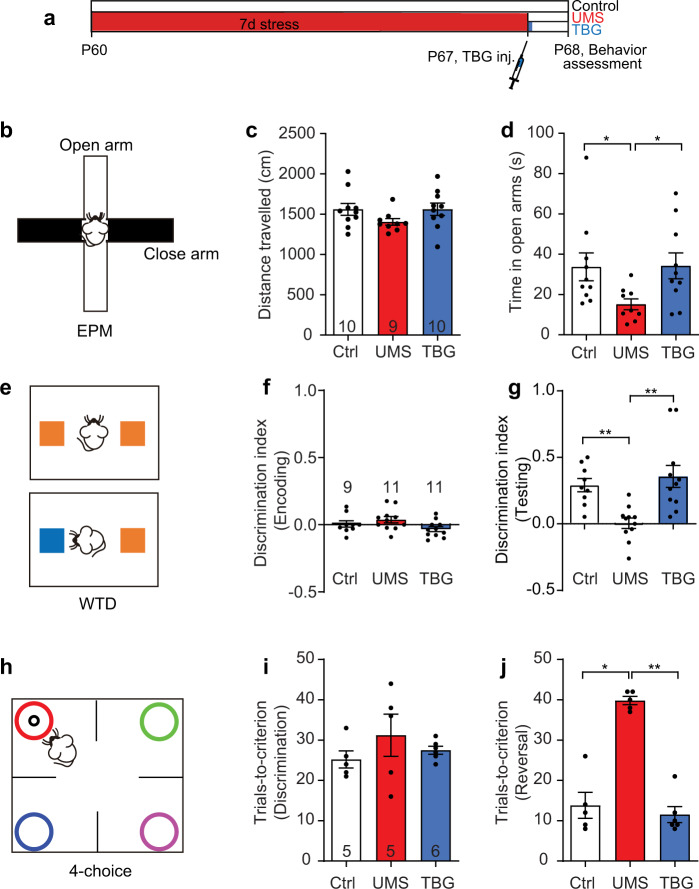
TBG rescues UMS-induced behavioral deficits in mice. **a** Timeline of UMS, drug injection, and behavioral tests. **b** Schematic of the elevated plus maze (EPM) test. **c** Total distance traveled in EPM (*H*(3) = 4.437, *p* = 0.1088, Kruskal–Wallis test). **d** Time spent in the open arms (*H*(3) = 8.275, *p* < 0.05, Kruskal–Wallis test with *post hoc* Dunn’s multiple comparisons test). **e** Schematic of the whisker-dependent texture discrimination (WTD) task. The two colors represent distinct textures. **f**, **g** Texture preference during encoding (*H*(3) = 5.250, *p* = 0.072, Kruskal–Wallis test) and testing (*H*(3) = 16.061, *p* < 0.001, Kruskal–Wallis test with *post hoc* Dunn’s multiple comparisons test). Discrimination index is defined as the number of approaches to one texture (e.g., novel) minus the number of approaches to the other texture (e.g., familiar), divided by the total number of approaches. **h** Schematic of the 4-choice odor discrimination and reversal task. Each color symbolizes a distinct odor; only one is associated with the food reward (black circle). **i**, **j** Number of trials taken to reach the performance criterion in the initial discrimination (*F*(2,13) = 0.8922, *p* = 0.4334, one-way ANOVA) and reversal phase (*H*(3) = 9.954, *p* < 0.01, Kruskal–Wallis test with *post hoc* Dunn’s multiple comparisons test). The performance criterion is 8 correct choices out of 10 consecutive trials. Hereinafter Ctrl stands for the unstressed control group, UMS stands for the stress group without post-stress TBG treatment, and TBG stands for the post-stress TBG treatment group; data shown as mean ± s.e.m.; *n* = number of mice unless otherwise indicated; *p < 0.05, **p < 0.01, ***p < 0.001, ****p < 1 × 10^−4^.

As uncertainty about the future is a major source of stress [59], the paradigm of unpredictable mild stress (UMS), in which different stressors are presented each day at random, well mimics the variability and stochasticity of real-life stress. We thus adopted 7-day UMS as the stress protocol (Supplementary Table 1) and conducted behavioral tests one day after the termination of UMS (Fig. 1a). First, we assessed the mouse’s anxiety level using the elevated plus maze (EPM, Fig. 1b), which measures the mouse’s exploratory behavior against its innate fear of height. Despite traveling similar distances (Fig. 1c), UMS mice spent less time in the open arms than controls (Fig. 1d), indicating a higher anxiety level. We then evaluated sensory processing with a whisker-dependent texture discrimination (WTD) task, which exploits the mouse’s innate preference for novelty [43] (Fig. 1e). Control and UMS mice exhibited comparable locomotion and exploratory behavior (Fig. 1f and Supplementary Fig. 1). However, while control mice preferred the novel texture, UMS mice lost such preference (Fig. 1g), suggesting a loss of the capability to distinguish the two textures [15]. Finally, we assessed cognitive flexibility with a four-choice odor discrimination and reversal task (“4-choice task”, Fig. 1h). This task evaluates the mouse’s flexibility in learning an odor-reward contingency and then reversing the association [42, 60]. While UMS mice performed comparably to control mice during the initial discrimination phase (Fig. 1i), they took significantly more trials to learn the new odor-reward contingency in the reversal phase (Fig. 1j), indicating reduced cognitive flexibility. Proceeding to evaluate TBG’s effects on stress-induced behavioral deficits, we injected a single dose of TBG (10 mg/kg) into the mice immediately after 7d UMS and conducted behavioral tests one day later. We chose this dosage as previous pharmacokinetic studies indicated that it was the lowest dose likely to lead to sufficient concentration in the brain to activate 5-HT_2_ receptors [36]. Excitingly, we found that TBG alleviated stress-induced anxiety (Fig. 1d), restored the novel texture preference in WTD (Fig. 1g and Supplementary Fig. 1), and normalized cognitive flexibility in the 4-choice task (Fig. 1j). In contrast, treatment with a single dose of fluoxetine (10 mg/kg), a selective serotonin reuptake inhibitor, failed to rescue the behavioral defect; saline treatment did not rescue it either (Supplementary Fig. 2). These findings demonstrate TBG’s fast effects in combating behavioral impairments induced by UMS.

### Post-stress TBG treatment promotes dendritic spine regrowth on cortical PyrNs

**Fig. 2:**
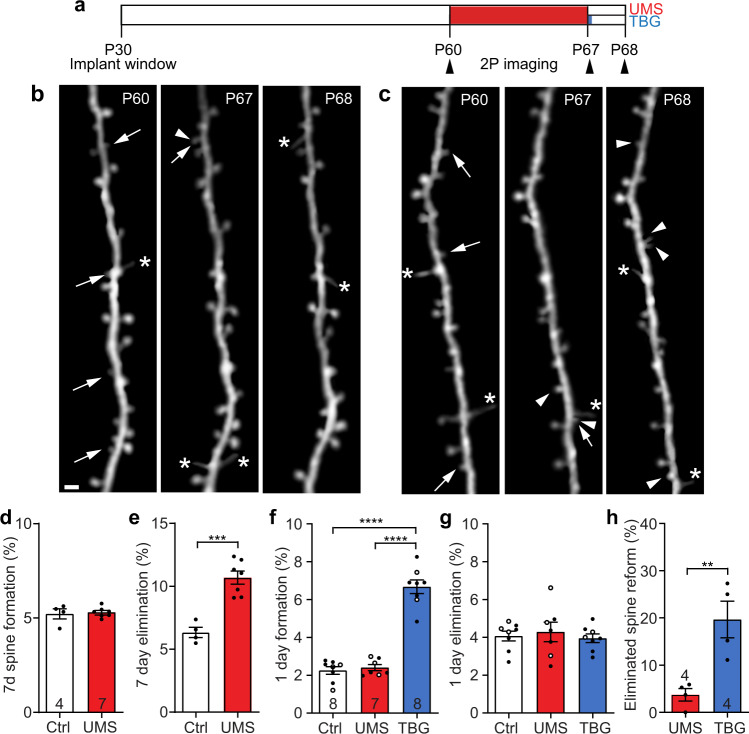
TBG promotes spine formation that partially compensates for UMS-induced spine loss in the mouse cortex. **a** Timeline of dendritic spine imaging experiments. **b** Example of the same set of S1BF spines imaged before UMS, immediately after UMS, and after 1-day recovery. **c** Example spine imaging over the same time course but with post-stress TBG treatment. Arrow: eliminated spine; arrowhead: new spine; asterisk: filopodium. Scale bar: 2 µm. **d**, **e** Spine formation (*t*(9) = 0.3430, *p* = 0.7395, unpaired *t*-test) and elimination (*t*(9) = 5.723, *p* < 0.001, unpaired *t*-test) over 7 days. Hereinafter filled circles represent data from S1BF and empty circles represent data from frontal cortex. **f**, **g** Spine formation (*F*(2,20) = 92.92, *p* < 1 × 10^−4^, one-way ANOVA with *post hoc* Tukey’s multiple comparisons test) and elimination (*F*(2,20) = 0.2358, *p* = 0.7921, one-way ANOVA) over 1 day in control and during post-UMS recovery. **h** Percentage of spines eliminated during UMS that re-emerged during recovery (*t*(6) = 3.895, *p* < 0.01, unpaired *t*-test).

Recent work demonstrates that the dissociative anesthetic ketamine boosts dendritic spine formation and rescues stress-induced behavioral deficits [61]. As TBG promotes dendritic growth and spine formation like classical psychedelics [36], we asked whether TBG could restore spines in the stressed brain. We followed spines on apical dendrites of cortical layer (L) 5 PyrNs in *thy1*-GFP-M mice before and after UMS by in vivo two-photon (2P) microscopy (Fig. 2a–c). We found that 7-day UMS increased spine elimination without affecting their formation (Fig. 2d, e). Importantly, post-stress TBG treatment almost doubled spine formation (Fig. 2f) within a day but did not alter spine elimination (Fig. 2g). More than 32% of such new spines emerged close to the site (less than 2 μm) where a spine had been lost previously, similar to the observation under ketamine treatment [61]. Such rapid increase in spine formation was not observed in UMS mice treated with fluoxetine or saline (Supplementary Fig. 3). We further classified newly formed spines into distinct morphological categories: mushroom, stubby, thin, and others. In UMS mice, mushroom, stubby, and thin morphology accounted for 28.6%, 28.6%, and 32.1% out of 28 new spines, respectively. Although TBG elevated overall spine formation, the relative percentage of new spines in each morphological category remained approximately unchanged: mushroom 22.5%, stubby 28.8%, and thin 31.5% out of 111 new spines. In addition, we found that the dynamics of filopodia (long, thin protrusions without bulbous heads) did not differ between control, UMS, and TBG mice (Supplementary Fig. 4). Importantly, TBG-induced spine formation compensated for ~20% of spine loss during UMS, about 5 times as much as in spontaneous recovery (Fig. 2h). Furthermore, we found that 49.1% of new spines (27/55 from 4 mice) formed within 1-day after TBG treatment survived over 2 days, and 29.1% of them survived over 12 days, slightly higher than the survival rate of new spines in control animals reported previously [47, 62]. In summary, TBG rapidly promotes spine formation and selectively restores lost spines in the post-stress cortex.

### Post-stress TBG treatment increases whisking modulation of mesoscopic neural activity and normalizes spontaneous Ca activity of cortical PyrNs

**Fig. 3:**
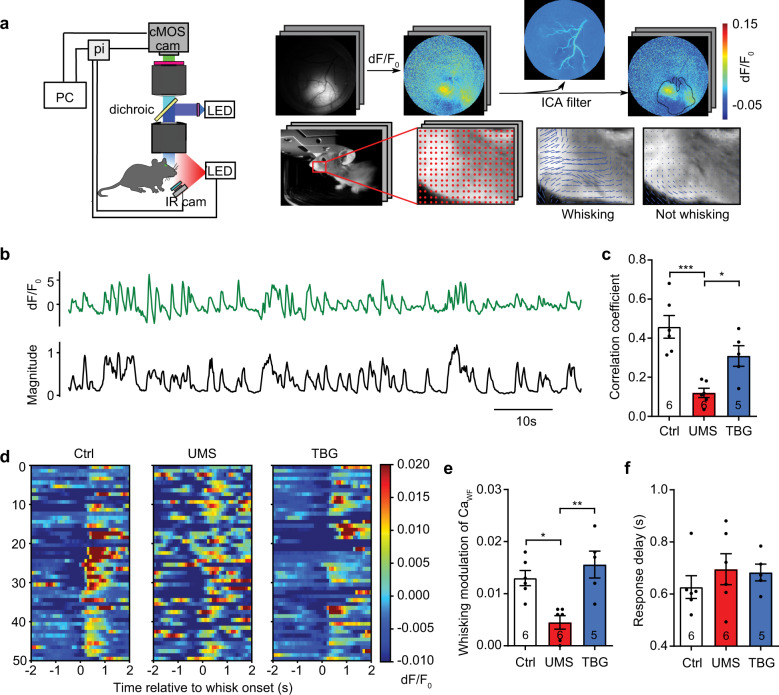
TBG normalizes baseline and whisking-modulation of mesoscopic neural activities in S1BF following UMS. **a** Schematic of mesoscope and data processing pipeline. pi: Raspberry Pi. Top: independent component analysis removes hemodynamic artifacts. Bottom: an optic flow algorithm identifies whisking episodes. **b** Representative traces of Ca_WF_ (green) and whisking magnitude (black) over time. **c** Correlation between Ca_WF_ and whisking magnitude (*F*(2,14) = 13.90, *p* < 0.001, one-way ANOVA with *post hoc* Tukey’s multiple comparisons test). **d** Examples of trial-by-trial Ca_WF_ time-locked to whisking onset. **e** Whisking-modulation of Ca_WF_ (*H*(3) = 11.36, *p* < 0.001, Kruskal–Wallis test with *post hoc* Dunn’s multiple comparisons test). **f** The response delay between whisking onset and the peak of Ca_WF_ (*H*(3) = 1.707, *p* = 0.4536, Kruskal–Wallis test).

As synaptic changes may alter neural network functions, we examined cortical neural activities with wide-field and 2P calcium (Ca) imaging. We focused on the mouse barrel cortex (S1BF) due to its well-established role in somatosensory processing and its optical accessibility. We labeled S1BF neurons with an adeno-associated virus (AAV) encoding the Ca indicator GCaMP6f driven by the human synapsin promoter. First, we performed wide-field Ca imaging using a custom-built mesoscope with simultaneous behavioral monitoring (Fig. 3a, Supplementary Movie 1). We found that the whole-field Ca activity (Ca_WF_, spatial average of dF/F_0_ across pixels) in control mice correlated well with whisking (Fig. 3b, c). Such correlation was reduced by UMS and rescued by TBG (Fig. 3c). Individual pixel’s dF/F_0_ also correlated with whisker movement (Supplementary Fig. 5a); the peak value of their cross-correlation was reduced after UMS and partially rescued by TBG (Supplementary Fig. 5b). To quantify the whisking-modulation of Ca_WF_, we measured the difference between the time average of Ca_WF_ after whisking onset (0.3s to 1.3s) and that immediately before whisking onset (−1s to 0s). UMS significantly decreased the whisking-modulation of Ca_WF_, which was completely rescued by TBG (Fig. 3d, e). In contrast, neither UMS nor post-stress TBG treatment changed the response delay (Fig. 3f).

**Fig. 4:**
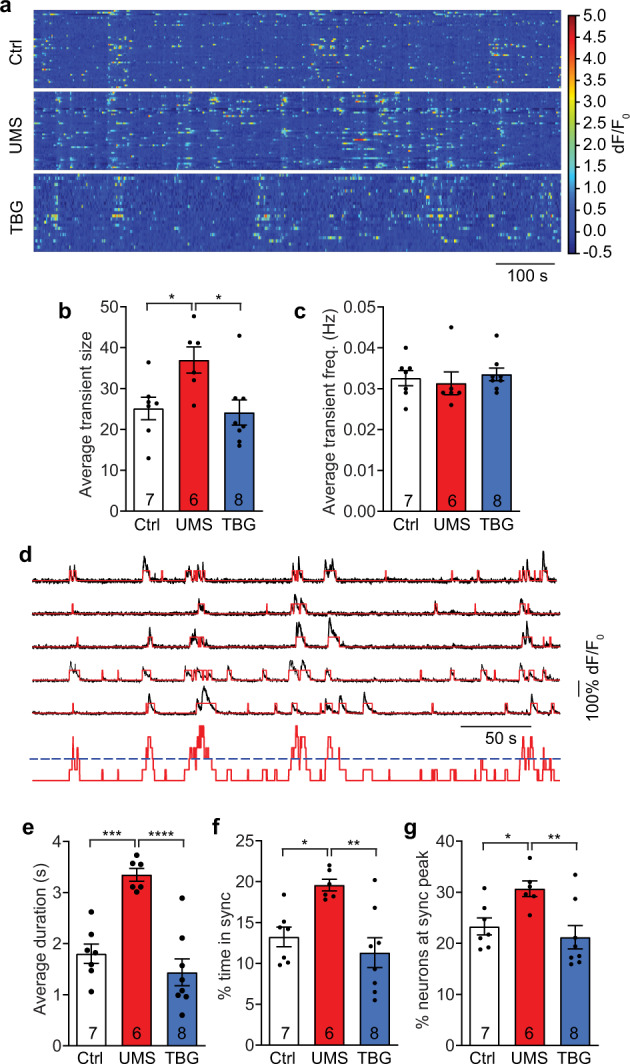
TBG normalizes baseline Ca activities of S1BF L2/3 neurons following UMS. **a** Example of baseline Ca activities of L2/3 neurons. **b** Average Ca transient size measured by the sum of dF/F_0_ (*F*(2,18) = 5.141, *p* < 0.05, one-way ANOVA with *post hoc* Tukey’s multiple comparisons test). **c** Average transient frequency (*H*(3) = 2.333, *p* = 0.3238, Kruskal–Wallis test). **d** Top: example of neurons with diverse activity levels and participating in synchronous events. Black: dF/F_0_; red: binarized Ca transients. Bottom: sum of binarized Ca transients (red) with threshold for synchrony (blue). **e** Average duration of synchronous events (*F*(2,18) = 20.75, *p* < 1 × 10^−4^, one-way ANOVA with *post hoc* Tukey’s multiple comparisons test). **f** Average percentage of time during which the neuronal population is in synchrony (*F*(2,18) = 8.473, *p* < 0.01, one-way ANOVA with *post hoc* Tukey’s multiple comparisons test). **g** Average percentage of neurons participating in synchronous events (*F*(2,18) = 6.202, *p* < 0.01, one-way ANOVA with *post hoc* Tukey’s multiple comparisons test).

We then performed 2P Ca imaging of L2/3 PyrNs in awake, head-fixed mice with cellular resolution (Fig. 4a). We found that overall Ca activity was significantly higher in UMS mice, shown as an increase in the average Ca transient size (i.e., sum of dF/F_0_ per transient, Fig. 4b) but not in transient frequency (Fig. 4c). Post-stress TBG treatment restored the Ca transient size to control level (Fig. 4b). To analyze the temporal pattern of neuronal activities, we quantified synchronous Ca events (Fig. 4d, Supplementary Fig. 6). The average duration of each synchronous event was longer after UMS and rescued by TBG (Fig. 4e). Likewise, UMS increased the average percentage of time the neuronal population was in synchrony and the average percentage of neurons participating in each synchronous event; TBG normalized both (Fig. 4f, g). These data suggest that TBG counteracts the effects of UMS on sensory cortex by decreasing the baseline neuronal activity and synchrony as well as enhancing the whisking-evoked neuronal responses, thus increasing signal-to-noise ratio for sensory input.

### TBG rescues the excitability of parvalbumin-expressing inhibitory interneurons (PV+ INs) in the stressed brain

Fast-spiking PV+ INs are the main source of inhibitory inputs onto PyrNs [63, 64]. They shape PyrN activity and synchrony as well as their sensory responses [65–68]. Previous studies have shown that intrinsic excitability of PV+ INs in deep layers of the cortex are decreased in mice subjected to restraint stress [15, 69]. To probe how UMS and TBG affect the intrinsic properties of PV+ INs, we performed patch-clamp recording of L2/3 PV+ INs from acute brain slices of PV-Cre;YFP^fl/fl^ mice. After UMS, the resting membrane potential became hyperpolarized and the input resistance decreased (Supplementary Fig. 7a, b), but the membrane capacitance, action potential (AP) threshold, and rheobase were unaffected (Supplementary Fig. 7c–e). TBG restored both resting potential and input resistance to the control level (Supplementary Fig. 7a, b), but interestingly decreased the rheobase (Supplementary Fig. 7e). Furthermore, after UMS the same injected current elicited fewer APs, and TBG restored AP firing to control levels (Supplementary Fig. 7f, g) Overall, in the stressed brain TBG increases the intrinsic excitability of PV+ INs, which may contribute to the altered baseline activity and synchrony of excitatory neurons in the S1BF.

### TBG restores the neuronal population with novel texture-specific response lost in the stressed brain

**Fig. 5:**
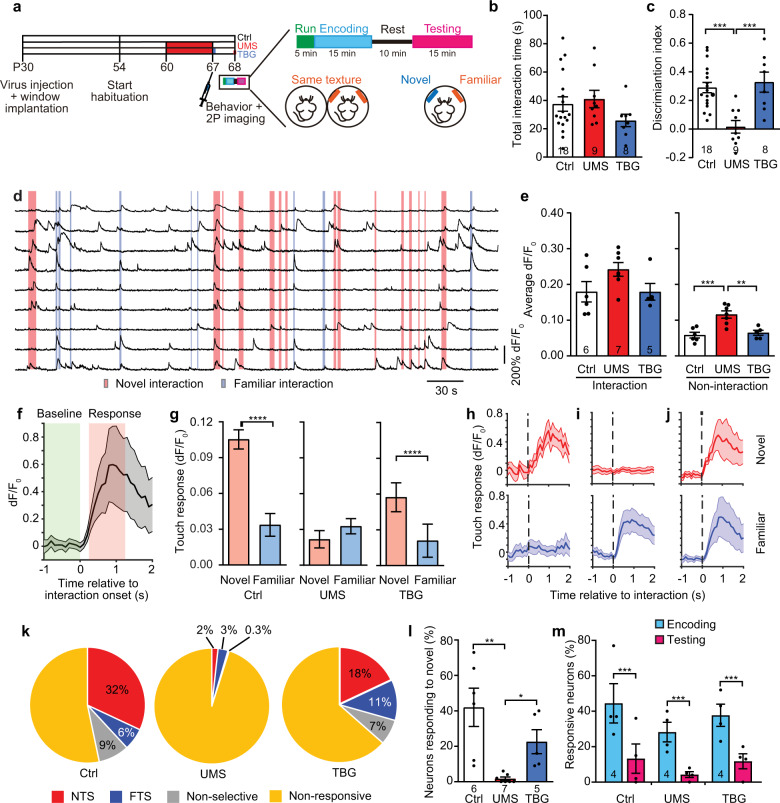
Ca activity of S1BF L2/3 neurons during texture interaction was altered by UMS and rescued by TBG. **a** Schematic of experimental design. **b** Total interaction time during testing (*F*(2,32) = 1.487, *p* = 0.2412, one-way ANOVA). **c** Discrimination index during testing (*F*(2,32) = 10.95, *p* < 0.001, one-way ANOVA with *post hoc* Tukey’s multiple comparisons test). **d** Examples of neuronal Ca traces from a control mouse. Shades: interaction with textures. **e** Average dF/F_0_ over the neuronal population during texture interaction (left; *H*(3) = 3.792, *p* = 0.1538, Kruskal–Wallis test) and non-interaction periods (right; *F*(2,15) = 13.47, *p* < 0.001, one-way ANOVA with *post hoc* Tukey’s multiple comparisons test). **f** Average Ca activity of an example neuron during texture interaction. Gray shades: s.e.m. **g** Average touch responses to novel or familiar texture. *n* = 244, 293, and 221 neurons for Ctrl, UMS, and TBG, respectively. Ctrl *W* = −17091, *p* < 1 × 10^−4^, UMS *W* = 2894, *p* = 0.3140, TBG W = −7787, *p* < 1 × 10^−4^; Wilcoxon signed-rank test for all. Examples of neurons responding to novel (**h**), familiar (**i**), or both (**j**) textures, shown as Ca activities aligned to interaction onset. Shades: s.e.m. **k** Categories of neurons based on the ROC analysis. **l** Percentages of neurons responding to the novel texture (*H*(3) = 13.03, *p* < 1 × 10^−4^, Kruskal–Wallis test with *post hoc* Dunn’s multiple comparisons test). **m** Percentages of neurons responding to the same texture presented during encoding and testing (two-way repeated measures ANOVA, effect of treatment: *F*(2,9) = 0.9776, *p* = 0.4129; effect of WTD phase: *F*(1,9) = 122.1, *p* < 1 × 10^−4^, *post hoc* Sidak multiple comparisons test).

Having examined TBG’s effects on the baseline and whisking-modulated neuronal activities in the stressed brain, we went on to study its effect on neuronal activities while the animal was engaged in sensory tasks. We adapted the WTD task for head-fixed mice using the Neurotar mobile home cage (MHC, Fig. 5a). We recorded mouse behavior with an infrared camera concurrently with 2P Ca imaging, annotated behavioral episodes (Supplementary Fig. 8), and characterized behavior-associated neuronal Ca activities. Despite the head-fixation and complete darkness, mice explored both textures as under the free-moving condition. We further showed that neither UMS nor post-stress TBG treatment altered total interaction time (Fig. 5b). However, UMS impaired texture discrimination and TBG rescued it (Fig. 5c), similar to the outcomes with free-moving mice. To characterize neuronal response to texture interaction, we compared their Ca activity when whiskers touched the texture with that during non-interaction periods (Fig. 5d, Supplementary Fig. 9). We found that neuronal activities in UMS mice were elevated, with significant change during non-interaction period, and a trend without significance during the interaction period; TBG restored neuronal activity during both periods back to the control level (Fig. 5e).

Next, we asked whether neurons respond to the novel and the familiar textures differently. We aligned Ca traces to the onset of texture interaction and calculated the touch response by subtracting the baseline activity from the activity after interaction onset (Fig. 5f). We found that in control mice, touch response to the novel texture was significantly higher than that to the familiar texture; this difference vanished after UMS but was restored by TBG (Fig. 5g). Furthermore, we performed receiver operating characteristic (ROC) analysis to categorize neurons (Fig. 5h-j). We found that in control mice 32% of neurons were novel texture-selective (NTS); in contrast, 6% of neurons were familiar texture-selective (FTS). Following UMS only 2% of neurons were NTS; the percentage of FTS neurons also dropped slightly (3%). After TBG treatment, the percentage of NTS neurons partially recovered (18%), and the percentage of FTS neurons even increased slightly (11%) (Fig. 5k). Taken together, the proportion of neurons that responded to the novel texture (NTS + non-selective) was dramatically reduced by UMS and rescued by TBG (Fig. 5l). Interestingly, when the same texture was presented during encoding and again during testing, the first time it elicited responses from a substantial fraction of neurons, but the second time much fewer neurons responded. This difference persisted in UMS and post-stress TBG treatment groups (Fig. 5m), suggesting that UMS did not abolish the reduction in neuronal responses after the texture had become familiar.

## Discussion

In this study we tested the rescuing effects of a single dose of TBG (10 mg/kg) administered after stress. A recent study examined the ability of a higher dosage of TBG (50 mg/kg) to impact spine growth and behavior mainly in unstressed mice [36]. Here, we expand on these findings to demonstrate that TBG can rescue UMS-induced deficits in dendritic spine structural dynamics, neuronal activities, and behavior. The efficacy of TBG at the lower dosage suggests that it has a broader therapeutic window than originally thought. It is also notable that behavioral rescuing is observed 1 day after TBG administration. In contrast, in human patients suffering from depression and anxiety disorders, symptomatic improvement with commonly prescribed selective serotonin reuptake inhibitors often starts after a week of use and gradually ramps up over a course of several weeks [70].

It is widely believed that the deleterious effects of chronic stress are due to allostatic overload [1]. TBG may exert its rescuing effects by increasing the allostatic capacity of the brain. We found that TBG promotes dendritic spine formation in both frontal and somatosensory cortices. The newly formed spines partially compensate for the spines lost during UMS. More importantly, being the postsynaptic sites of excitatory synapses, the formation of new spines, which reflects the addition of new synapses [71], provides a substrate for experience-dependent reorganization of neural circuits, which may tune circuits to better cope with the subsequent behavioral tasks [72]. Ketamine, an NMDA receptor antagonist, similarly enhances spine formation [73, 74], which is postulated to underlie its fast-acting antidepressant effect [61], although its primary target differs from that of psychedelics.

Human and rodent studies have demonstrated that psychological stress adversely affects sensory processing [75–77]. Our study shows that UMS increases the average neuronal activities at the baseline but not during texture contact, which may in effect diminish the signal-to-noise ratio of the neural signal. With the Neurotar mobile home-cage, we were able to conduct 2P Ca imaging while the head-fixed mouse performed a novel texture discrimination task. We found that UMS diminished the novelty-dependent response of S1BF neurons during texture interactions. As these neurons receive both bottom-up sensory inputs and top-down modulation from other cortical regions [78–80], the novelty-dependent response may reflect the top-down modulation of sensory information [81, 82]. During each encounter with a texture, the mouse not only notices its presence and detects its features [83–85], but also determines whether it has been encountered before. Our data suggest that if the texture is deemed familiar, top-down control suppresses activity in S1BF, presumably to minimize the redundant neural computation. Thus, the ability to differentiate between textures and the ability to perceive a texture as “novel” are decoupled. Stress impairs the former without abolishing the latter. The stressed mouse deems novel and familiar textures as identical, which elicits the suppression of S1BF activities, as evidenced by the dramatic drop in the percentage of NTS neurons to the level of FTS neurons.

Cortical L2/3 neurons are embedded in complex neural circuits. In addition to the canonical thalamus -> L4 -> L2/3 projections, S1BF L2/3 excitatory neurons receive cortical L5 inputs as well as direct inputs from the posterior medial thalamus (POm) or the ventral posteromedial nucleus (VPM) of the thalamus. Other long-range afferents may originate from non-whisker S1, whisker S2, and whisker M1 regions [86–88]. Therefore, the activity of L2/3 neurons may be modulated by both somatosensory and motor signals. Indeed, a previous study [53] suggests that the activity of S1BF L2/3 neurons increases during running (accompanied by whisking), a phenomenon we also observed. In addition, L2/3 neurons receive local recurrent excitation, which may amplify input-specific signals [89]. They also receive feedforward inhibition via PV+ INs, which may underlie their sparse firing [90]. As TBG is delivered systemically, it likely achieves its rescuing effects by acting on both local and distant circuits.

Our observation that TBG promotes spine formation on L5 PyrNs is consistent with the spinogenesis-promoting effect of psychedelics such as LSD, 2,5-dimethoxy-4-iodoamphetamine (DOI), and DMT, as well as non-psychedelic *N*,*N*-dimethylaminoisotryptamine (isoDMT) analogs, observed in vitro [91, 92]. As is well-known, classical psychedelics act through 5-HT_2A_ receptors, but they also have complex pharmacological properties [93]. For example, the hallucinogenic psychedelic LSD and the structurally similar non-hallucinogenic lisuride both act on 5-HT_2A_ receptors, but elicit distinct downstream intracellular signaling, which, together with their differential interaction profiles with other receptors (e.g., dopamine and 5-HT_1A_ receptors), may underlie the distinct transcriptomic, electrophysiological, and behavioral effects [37, 94]. As for TBG, previous screening across ~80 neuroreceptors revealed its high selectivity for 5-HT_2_, 5-HT_1B_, and α2A adrenergic receptors with additional affinity for the serotonin transporter (SERT) and monoamine oxidase A (MAO-A). Furthermore, it has been shown that the 5-HT_2A_ receptor antagonist ketanserin blocks TBG’s effect on neural plasticity and its antidepressant potential [36]. Given that ketanserin can block the therapeutic effects of TBG and has only weak to modest affinities for 5-HT_1B_ and α2A adrenergic receptors, it is likely that TBG exerts its effects on spine growth and behavior through activation of 5-HT_2_ receptors. A previous study [95] found the expression of 5-HT_2A_ receptors in cortical L5 PyrNs as well as middle-layer PV+ INs. This is consistent with our findings that TBG promotes spine formation on L5 PyrNs and increases the excitability of S1BF PV+ INs in the stressed brain. However, we cannot completely rule out the modulation by SERT and MAO-A. Thus the pharmacological targets for TBG’s therapeutic effects remain to be elucidated.

While TBG does not produce behavioral responses characteristic of serotonergic hallucinogens in rodents (e.g., the head-twitch response), only human clinical studies can ultimately confirm that it is non-hallucinogenic. Nevertheless, our work highlights the potential of using analogs of psychedelics to treat stress-related brain disorders. Furthermore, human clinical trials suggest that classical psychedelics such as LSD and psilocybin may have long-lasting therapeutic effects [96]. Our data show that TBG promotes rapid spine formation and slightly elevates their rate survival, leading to more newly formed spines being consolidated into the neuronal network. The functional implication of such effects remains to be elucidated. With the possibility of being developed into take-home medicines to facilitate patient access, this novel class of neuroplasticity-promoting (i.e., psychoplastogenic) compounds possess significant advantages over classical psychedelics [97].

## Supplementary information

Supplemental Material

Supplemental Movie 1

## Data Availability

Custom-written Python and Matlab codes are available upon request.
