# Supplementary material for: An analog of psychedelics restores functional neural circuits disrupted by unpredictable stress
Source: Mol Psychiatry. 2021 May 25;26(11):6237–52. doi: 10.1038/s41380-021-01159-1 (PMC8613316; doi:10.1038/s41380-021-01159-1)
Supplement: Supplementary file 1 — Supplemental Material [file 41380_2021_1159_MOESM1_ESM.docx]

An Analog of Psychedelics Restores Functional Neural Circuits Disrupted by Unpredictable Stress

Ju Lu, Michelle Tjia, Brian Mullen, Bing Cao, Kacper Lukasiewicz, Sajita Shah-Morales, Sydney Weiser, Lindsay P. Cameron, David E. Olson, Lu Chen, Yi Zuo

Supplementary Table 1: Protocol of 7-day UMS.

| Day | Light Cycle | | Dark Cycle |
| --- | --- | --- | --- |
|  | First half | Second half |  |
| 1 | Restraint stress 30 min | Restraint stress 30 min | Home cage space reduction |
| 2 | Exposure to a new room 30 min +  orbital shaker 30 min | Exposure to loud sudden noise 5 times +  tail suspension 6 min | Wet bedding |
| 3 | Exposure to new mice | | Light exposure |
| 4 | Social isolation | | Tilted cage |
| 5 | Tilted cage | Island isolation | No bedding |
| 6 | No bedding | No bedding + random air puff 5-10 times | Foreign objects |
| 7 | Foreign objects | Food deprivation | Food deprivation + continuous exposure to loud music |

**Supplementary Figures**

**
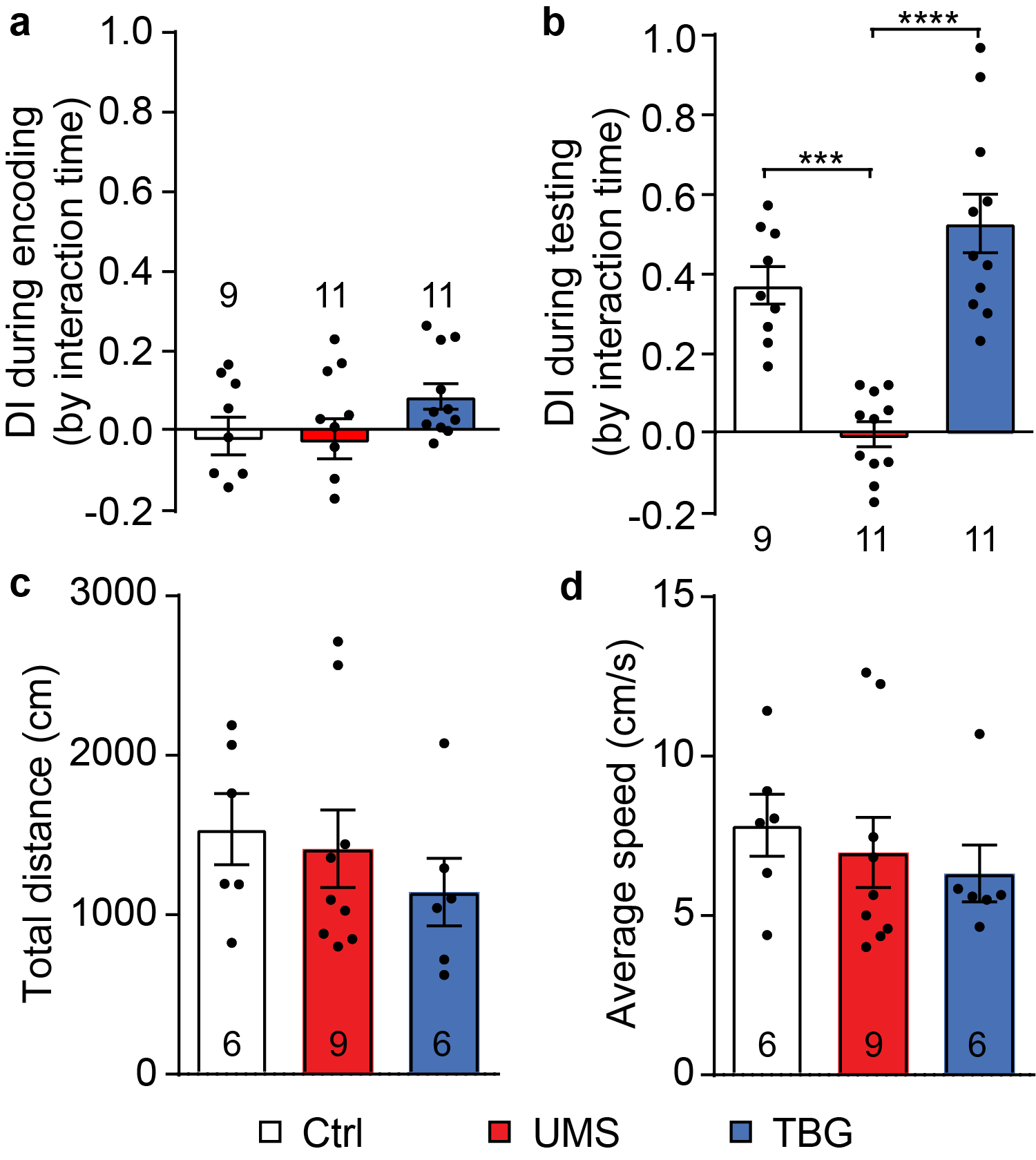
**

**Supplementary Figure 1: Effects of UMS and post-stress TBG treatment on texture discrimination and locomotion. a,b,** Measuring texture preference by interaction time leads to the same conclusions as measurement by interaction number: neither UMS nor post-stress TBG treatment affects texture exploration during encoding (*H*(3) = 2.737, *p* = 0.2545, Kruskal-Wallis test), but UMS impairs the texture discrimination ability and TBG rescues it (*F*(2,28) = 26.421, *p* < 1×10^-4^, one-way ANOVA with *post hoc* Tukey’s multiple comparisons test). **c,d,** Neither UMS nor TBG affects the total running distance (*H*(3) = 1.583, *p* = 0.4700, Kruskal-Wallis test) or the running speed (*H*(3) = 1.554, *p* = 0.4778, Kruskal-Wallis test) during testing. *n* = number of mice.

**
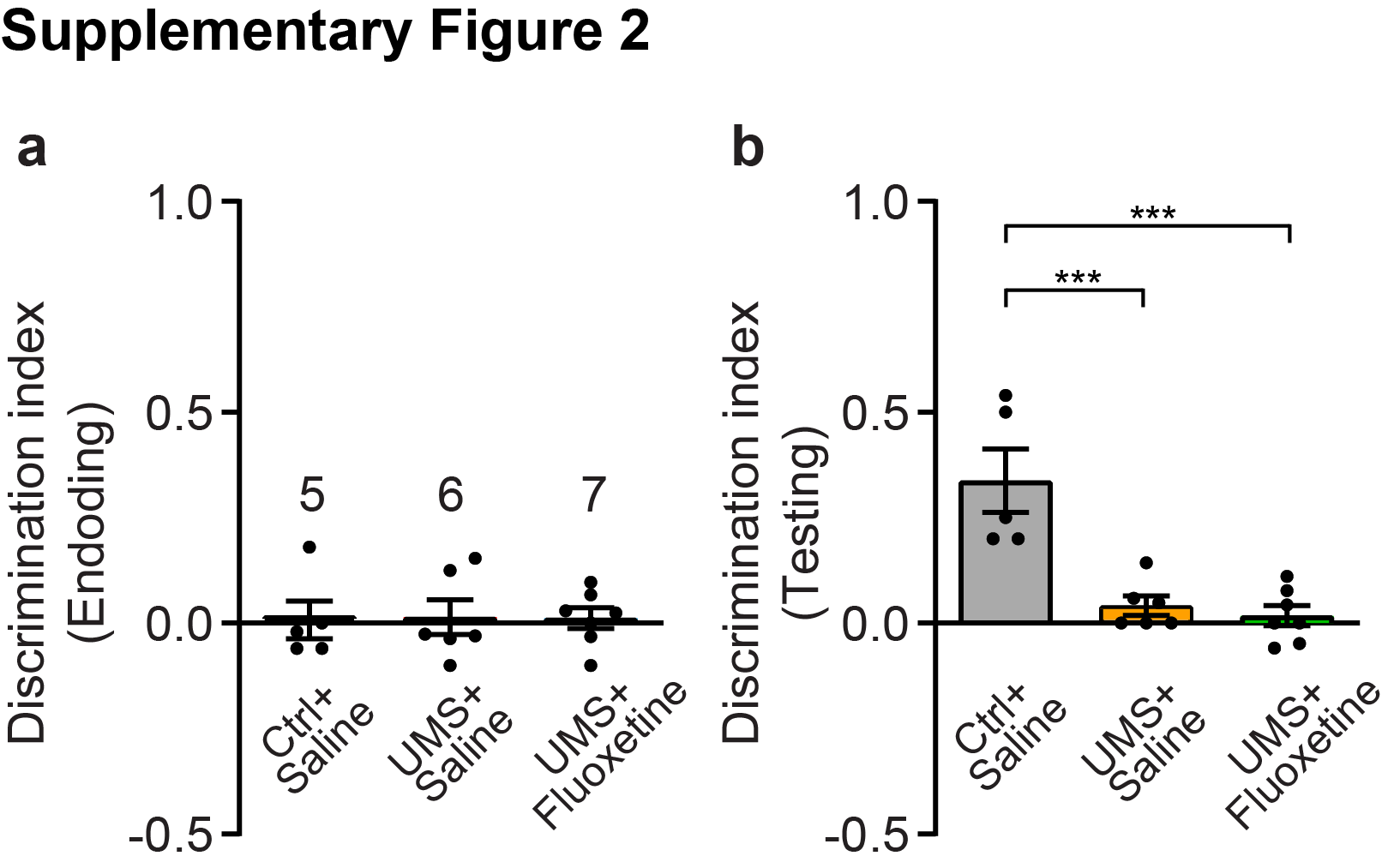
**

**Supplementary Figure 2: Effects of vehicle and fluoxetine treatment on WTD performance. a,** Lack of column preference during the encoding phase among all three experimental groups (*H*(3) = 0.2521, *p* = 0.8895, Kruskal-Wallis test). **b,** Neither saline nor fluoxetine treatment restored novel texture preference in UMS mice (*F*(2,15) = 17.09, *p* < 0.001, one-way ANOVA and *post hoc* Tukey’s multiple comparisons test). *n* = number of mice.

**
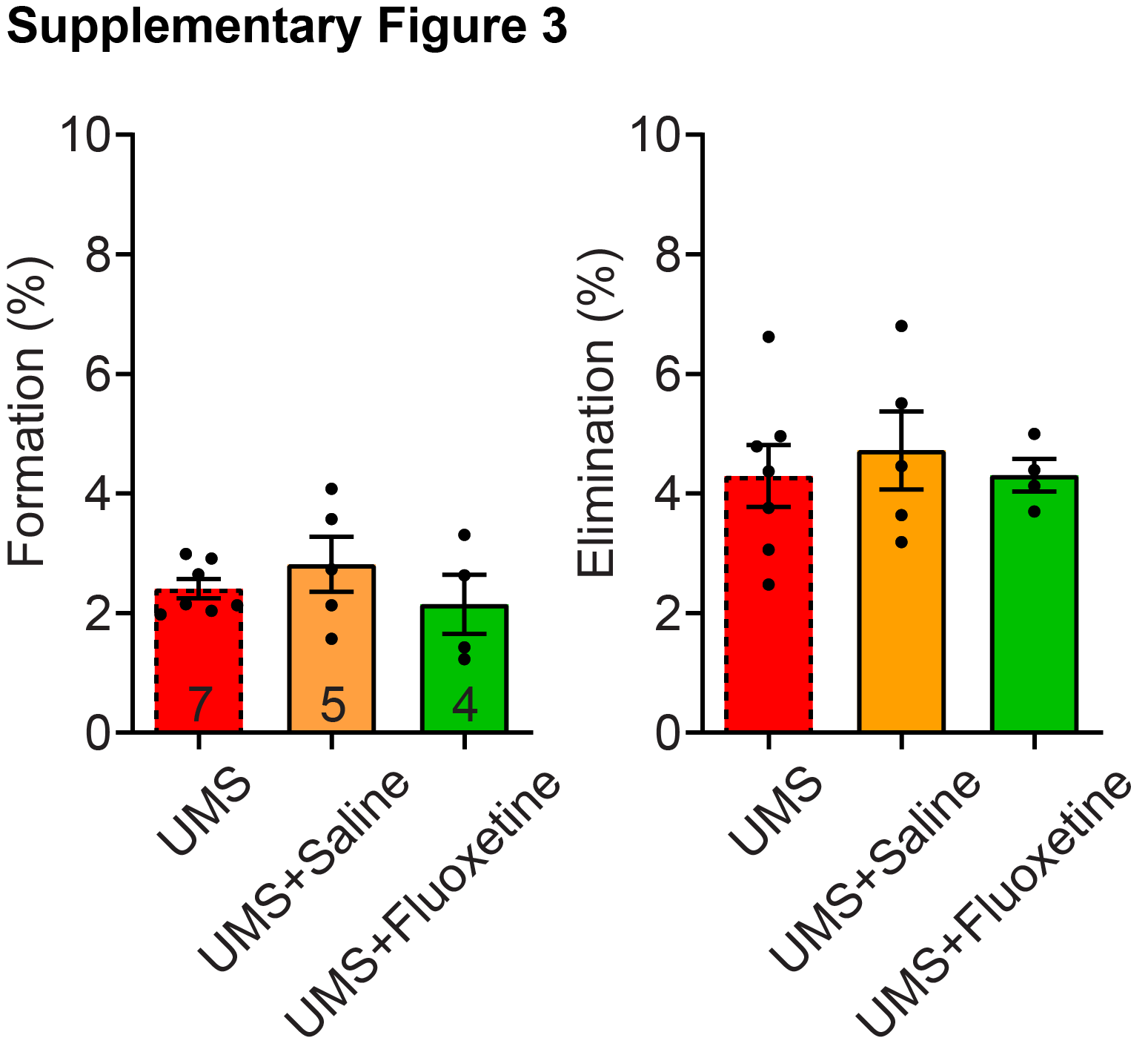
**

**Supplementary Figure 3: Neither fluoxetine nor vehicle treatment affects spine dynamics in UMS mice.** All three groups of mice exhibited comparable spine formation (*F*(2, 13) = 0.8162, *p* = 0.4635, one-way ANOVA) and elimination (*F*(2, 13) = 0.1947, *p* = 0.8255, one-way ANOVA) over 1-day. UMS data the same as in Figure 2f and 2g. *n* = number of mice.

**
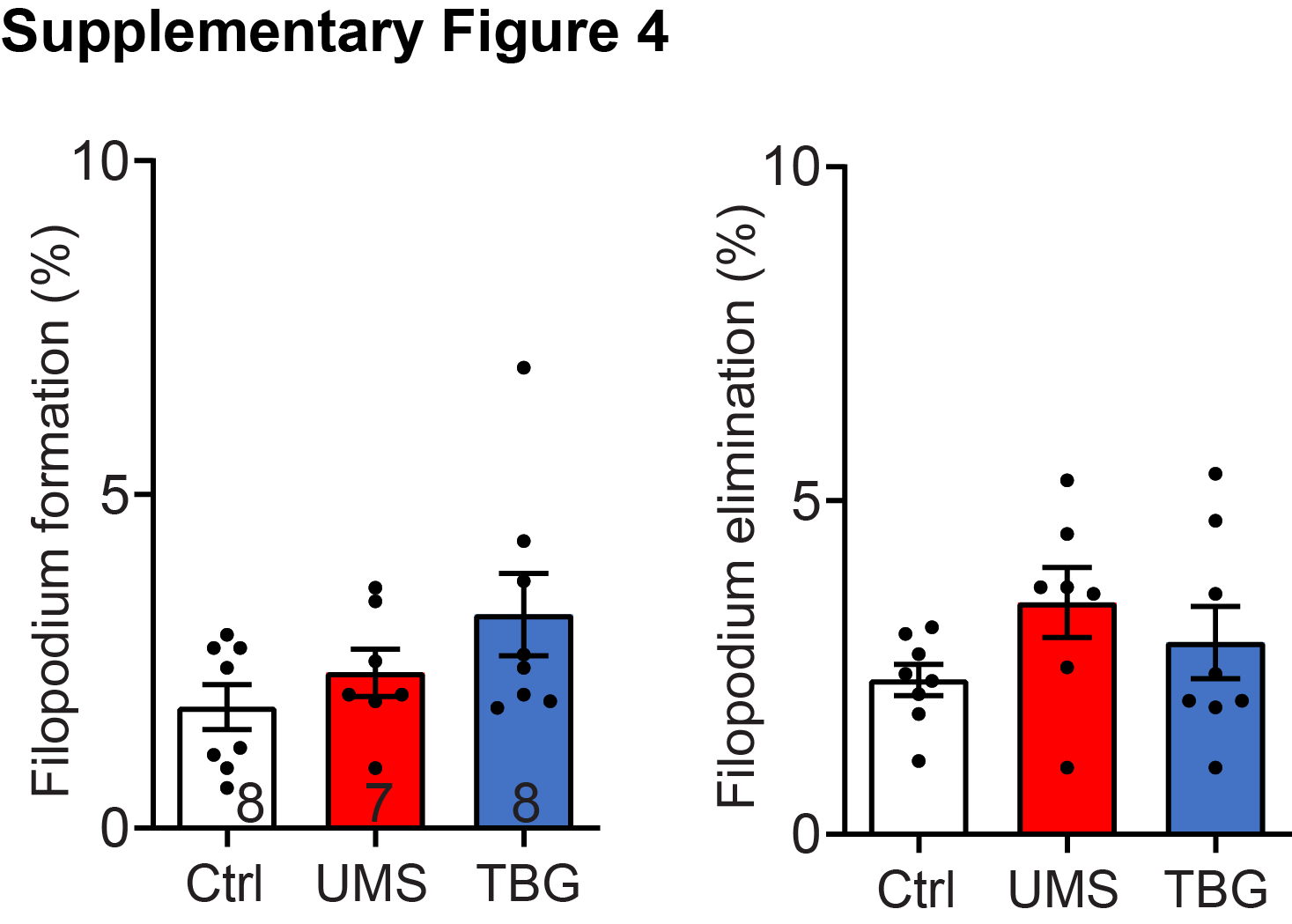
**

**Supplementary Figure 4: Dynamics of filopodia is not altered by UMS or TBG.** All three groups of mice exhibited comparable filopodia formation (*H*(3) = 2.298, *p* = 0.3170, Kruskal-Wallis test) and elimination (*F*(2, 20) = 1.615, *p* = 0.2237, one-way ANOVA) over 1 day. *n* = number of mice.

**
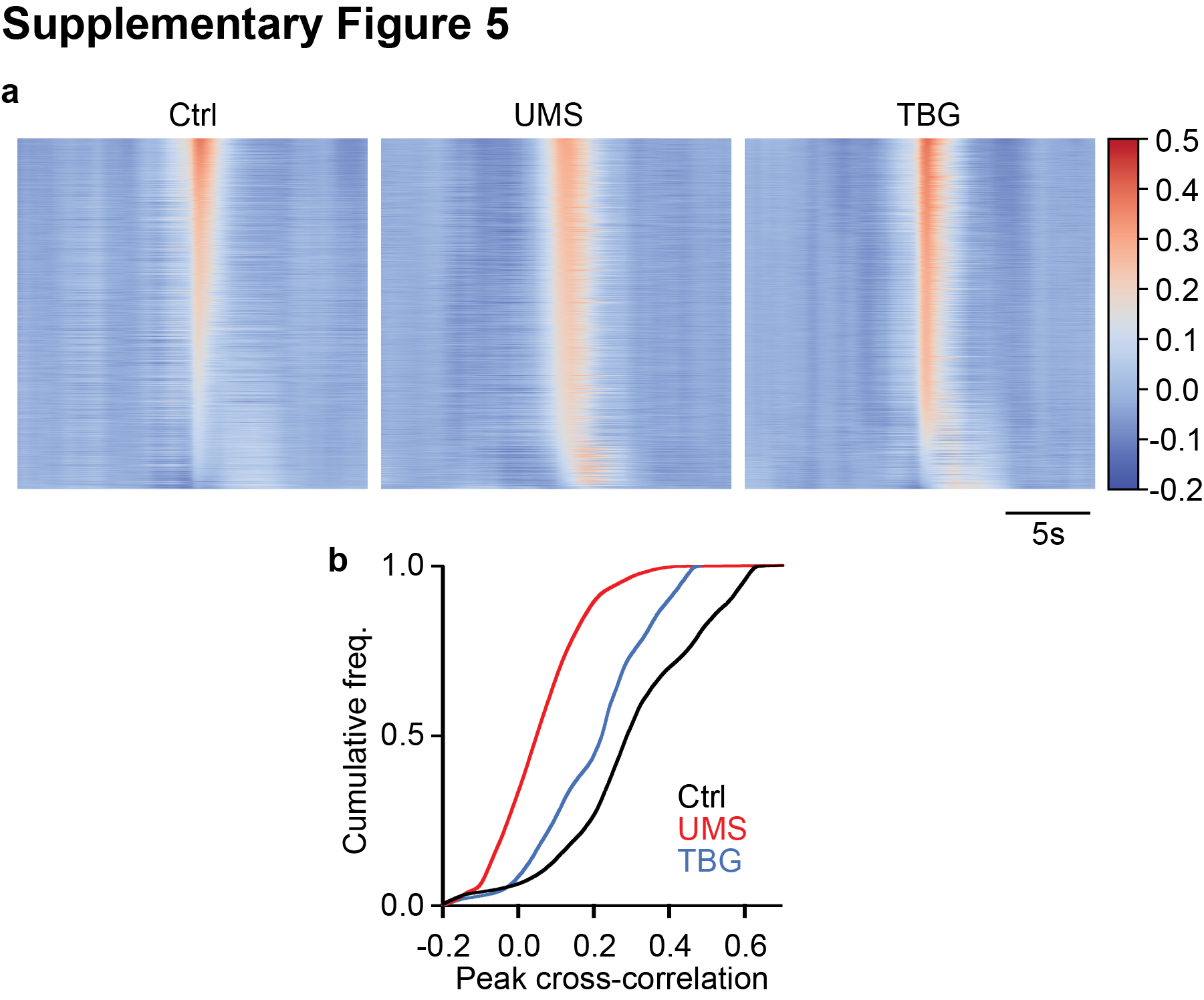
**

**Supplementary Figure 5: Pixel-wise correlation between mesoscopic Ca signals and whisking magnitude. a,** Cross-correlation between individual pixel’s dF/F_0_ and whisking magnitude over time lags of (-10s, 10s) in a representative mouse before UMS, after UMS, and after post-stress TBG treatment. Color bar: Pearson correlation coefficient. **b,** Cumulative probability distribution curves of the peak values of pixel-wise cross-correlation. Data pooled over all mice in Fig. 3E and F. *n* = 50859, 99634, and 25542 for Ctrl, UMS, and TBG groups, respectively; *p* < 1×10^-4^ for all comparisons between conditions, Kolmogorov-Smirnov test.

**
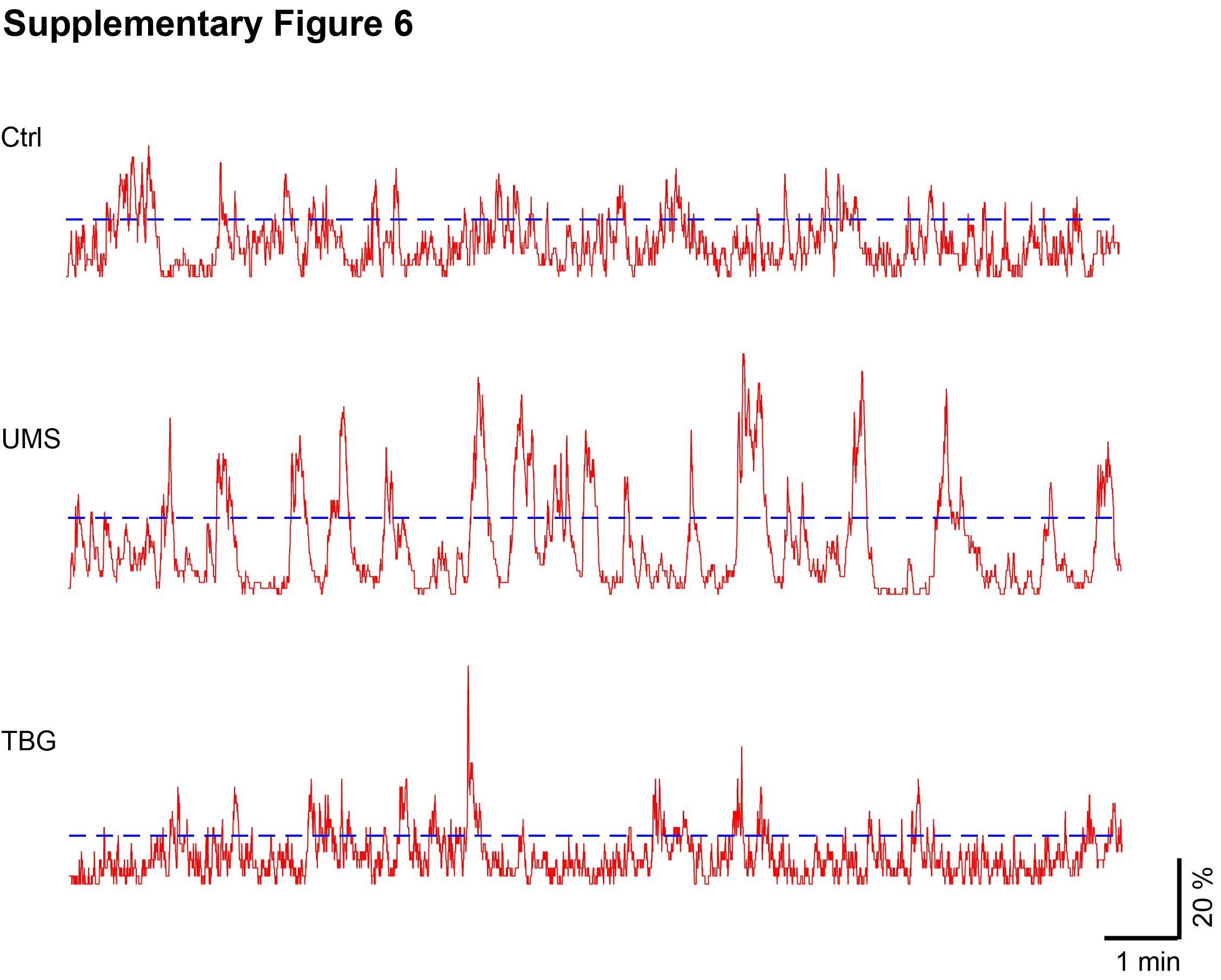
Supplementary Figure 6: Examples of synchronous neuronal activities in control, UMS, and TBG mice.** Red: sum of binarized Ca transients of all cells. Dotted blue lines: threshold for synchrony. Vertical scale bar: percentage of synchronously active neurons. Number of neurons: 71 (Ctrl), 69 (UMS), and 50 (TBG).

**
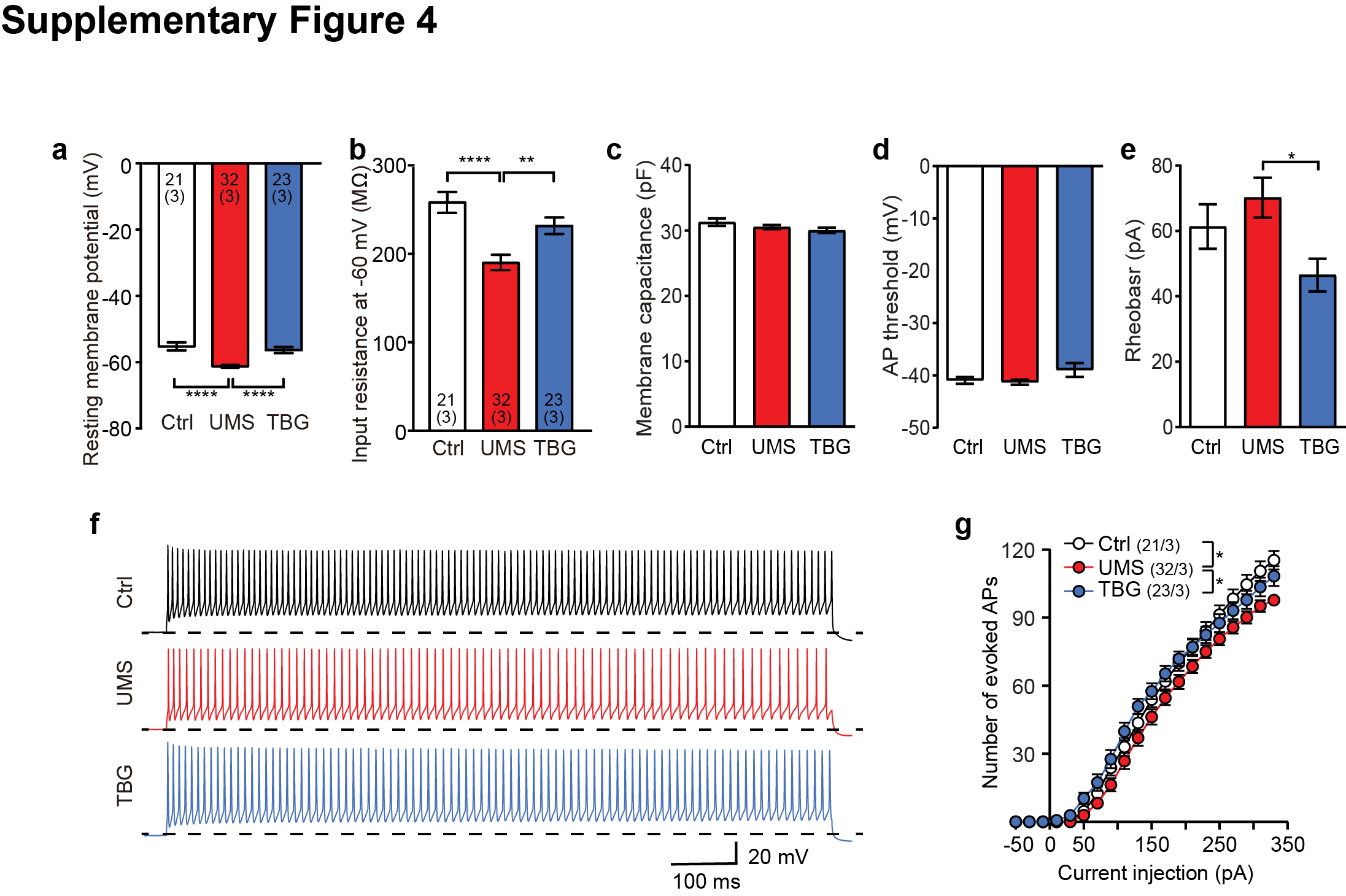
Supplementary Figure 7: TBG rescues PV+ IN electrophysiological properties perturbed by UMS.** Quantification of the resting membrane potential (**a**, *H*(3) = 29.18, *p* < 1×10^-4^, Kruskal-Wallis test with *post hoc* Dunn’s multiple comparisons test), input resistance at -60 mV holding voltage (**b**, *H*(3) = 20.18, *p* < 1×10^-4^, Kruskal-Wallis test with *post hoc* Dunn’s multiple comparisons test), membrane capacitance (**c**, *F*(2,73) = 1.968, *p* = 0.1471, one-way ANOVA), action potential threshold (**d**, *F*(2,73) = 2.234, *p* = 0.1144, one-way ANOVA), and Rheobase (**e**, *H*(3) = 7.194, *p* < 0.05, Kruskal-Wallis test with *post hoc* Dunn’s multiple comparisons test) of PV+ INs in control, UMS, and UMS+TBG mice. **f,** Sample traces in response to a 330 pA step current injection from control, UMS, and UMS+TBG mice. Dashed line: -60 mV. **g**, Input-output relationship between the total number of APs and the step current injections (two-way repeated measures ANOVA, main effect of treatment: Ctrl *vs.* UMS *F*(1,51) = 4.797, *p* < 0.05; UMS *vs.* TBG *F*(1,53) = 5.627, *p* < 0.05; Ctrl *vs.* TBG *F*(1,42) = 0.015, *p* = 0.9021). *n* = number of neurons; number of mice per experimental group given in parenthesis.


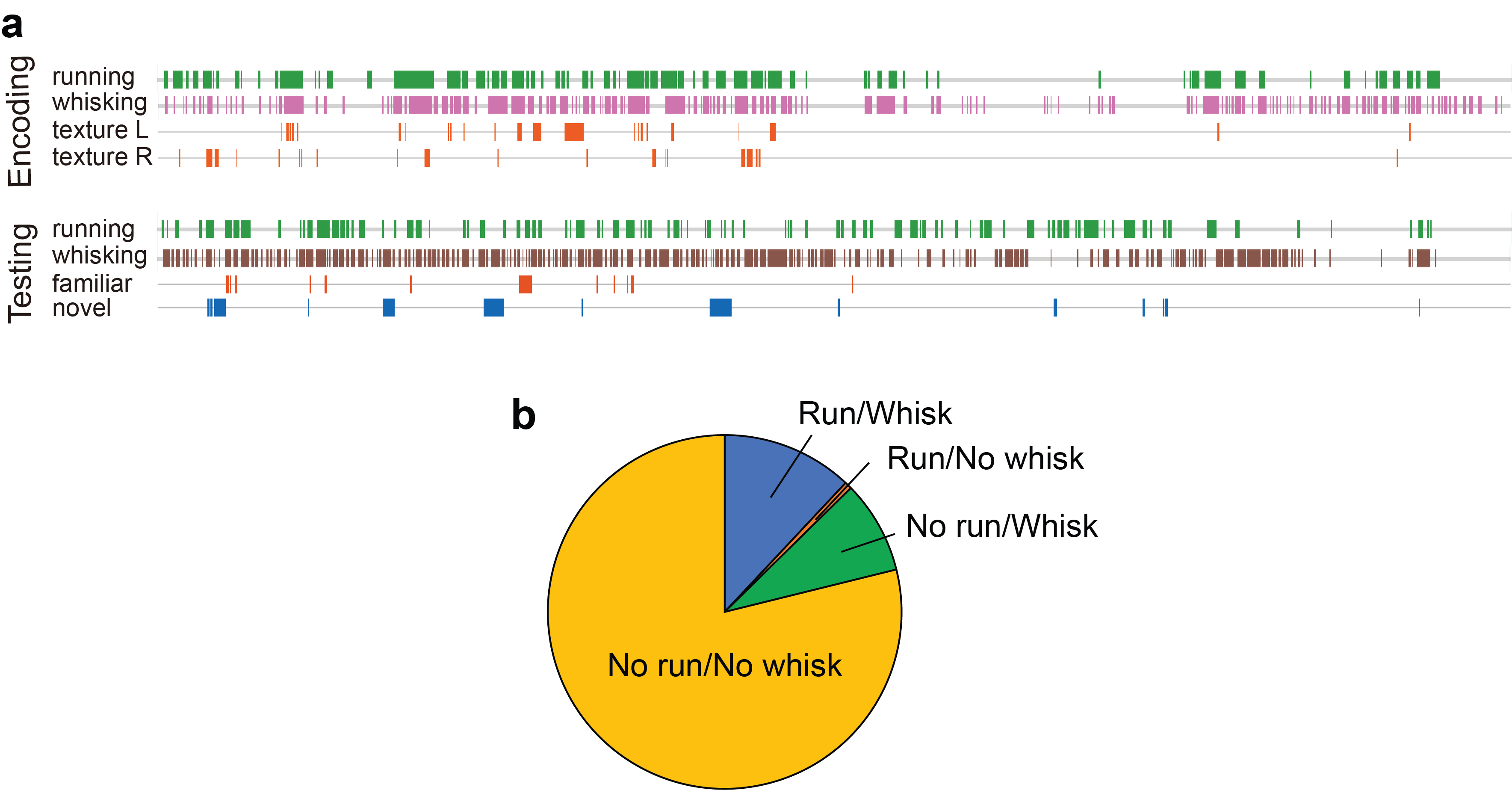
Supplementary Figure 8. Characterization of mouse running and whisking on Neurotar MHC. a, Example of annotated control mouse behavior during encoding and testing. b, Average percentage of time spent on different behaviors by control mice. *n* = 6 mice.


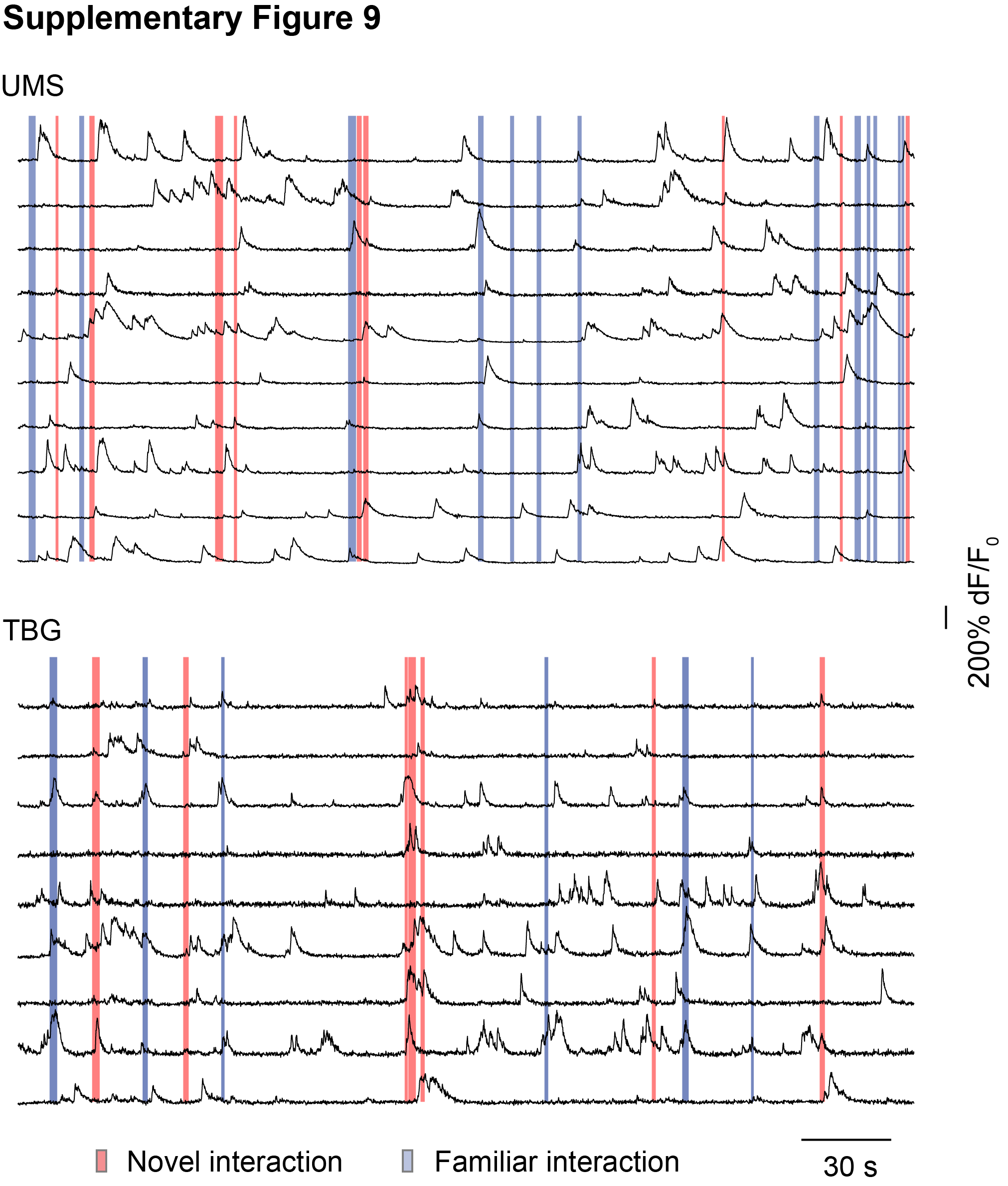
Supplementary Figure 9. Examples of activities of selected neurons time-locked to texture interactions in a UMS and a TBG mouse. Shades: interaction with textures.

Supplementary Movie 1. Example of mesoscopic Ca imaging with whisking behavior monitoring. Top: mouse whisking behavior monitored with IR camera (left) synchronized with wide-field Ca imaging (right). Color bar: dF/F_0_. Bottom: whisking magnitude (left) synchronized with the wide-field Ca activity Ca_WF_ (right).
